# Exosome-Contained APOH Associated With Antiphospholipid Syndrome

**DOI:** 10.3389/fimmu.2021.604222

**Published:** 2021-05-04

**Authors:** Yuan Tan, Yiding Bian, Yunfeng Song, Qinhua Zhang, Xiaoping Wan

**Affiliations:** ^1^ Department of Integrated Traditional Chinese Medicine (TCM) & Western Medicine, Shanghai First Maternity and Infant Hospital, School of Medicine, Tongji University, Shanghai, China; ^2^ Department of Gynecology, Shanghai First Maternity and Infant Hospital, School of Medicine, Tongji University, Shanghai, China

**Keywords:** antiphospholipid syndrome, exosomes, proteomics analysis, mice model, MAPK pathway

## Abstract

**Background:**

Antiphospholipid syndrome (APS) is a systemic autoimmune disease that can lead to thrombosis and/or pregnancy complications. Exosomes, membrane-encapsulated vesicles that are released into the extracellular environment by many types of cells, can carry signals to recipient cells to affect angiogenesis, apoptosis, and inflammation. There is increasing evidence suggesting that exosomes play critical roles in pregnancy. However, the contribution of exosomes to APS is still unknown.

**Methods:**

Peripheral plasma was collected from healthy early pregnancy patients (NC-exos) and early pregnancy patients with APS (APS-exos) for exosome extraction and characterization. The effect of exosomes from different sources on pregnancy outcomes was determined by establishing a mouse pregnancy model. Following the coincubation of exosomes and human umbilical vein endothelial cells (HUVECs), functional tests examined the features of APS-exos. The APS-exos and NC-exos were analyzed by quantitative proteomics of whole protein tandem mass tag (TMT) markers to explore the different compositions and identify key proteins. After incubation with HUVECs, functional tests investigated the characteristics of key exosomal proteins. Western blot analysis was used to identify the key pathways.

**Results:**

In the mouse model, APS-exos caused an APS-like birth outcome. In vitro experiments showed that APS-exos inhibited the migration and tube formation of HUVECs. Quantitative proteomics analysis identified 27 upregulated proteins and 9 downregulated proteins in APS-exos versus NC-exos. We hypothesized that apolipoprotein H (APOH) may be a core protein, and the analysis of clinical samples was consistent with finding from the proteomic TMT analysis. APOH-exos led to APS-like birth outcomes. APOH-exos directly enter HUVECs and may play a role through the phospho-extracellular signal-regulated kinase pathway.

**Conclusions:**

Our study suggests that both APS-exos and APOH-exos impair vascular development and lead to pregnancy complications. APOH-exos may be key actors in the pathogenesis of APS. This study provides new insights into the pathogenesis of APS and potential new targets for therapeutic intervention.

## Introduction

Antiphospholipid syndrome (APS) is a systemic autoimmune disease characterized by thrombosis and/or pregnancy complications due to persistent (≥12 weeks) medium/high titers of antiphospholipid antibodies (aPLs) (1, 2). Catastrophic APS may lead to widespread small-vessel thrombosis and multiorgan failure, with a mortality rate of more than 50% (3). APLs, including IgG and IgM of anti-b2-glycoprotein I antibodies (also known as anti-b2-GPI; anti-APOH) and/or cardiolipin (aCL), and /or lupus anticoagulant, which have a clear correlation with the clinical manifestations of APS, but the pathophysiology between them is not clear (1, 4). The major target of aPLs is apolipoprotein H (APOH, also known as β2-glycoprotein I, β2GPI), a plasma protein that binds avidly to phospholipid surfaces, especially when dimerized by binding to aPLs (5–9). Endothelial dysfunction is a key pathological component of APS (10). The pathological manifestations of APS are vascular remodeling, angiogenesis injury and endothelial cell injury. Long-term anticoagulant therapy is the only potent treatment for APS. However, it is not effective in all patients and increases the risk of bleeding (5). There is an urgent need to explore the etiology of APS.

Exosomes are saucer-like vesicles with diameters of approximately 30–150 nm. They are released from various cell types, such as fetal cells, immunocytes and tumor cells (10–12). Studies have suggested that exosomes and their contents (i.e., proteins, mRNAs, and microRNAs) contribute to the regulation of multiple biological processes during pregnancy (12, 13). Accumulating evidence supports the hypothesis that exosomes play significant roles in the pathophysiology of inflammation, thrombosis and autoimmune diseases (14, 15). In the pathology of APS, exosomes may injure the vascular endothelium function as an independent mechanism. (16). To date, little is known about the role exosomes play in APS during pregnancy.

In this study, we provide novel evidence that APS-exosomes (exos) and APOH-exos are associated with adverse reproductive events in a mouse model, likely facilitated by impaired endothelial cell functions.

## Materials and Methods

### Ethics Statement

From July 2018 to December 2018, plasma samples from healthy pregnant women and pregnant women with APS were collected at the First Maternal and Infant Health Hospital Affiliated with Tongji University (n = 6 per group, 12 in total). Three umbilical cords were obtained from healthy newborns at the same hospital. Approval was obtained from the Institutional Ethics Committee of the Shanghai First Maternal and Infant Hospital affiliated with Tongji University (KS18128). All mothers provided written informed consent and were of the same age and gestational age ranges.

The current study used plasma (time of sampling was between 6–8 weeks of gestation). Blood was drawn into vacutainer tubes containing sodium citrate (0.129 M). Plasma was obtained by centrifugation of the blood at 3000 × g for 5 min at 4°C and frozen at −80°C (Thermo Scientific, USA) until analysis.

### Extraction of Exosomes by Ultracentrifugation

Exosomes were initially extracted from the plasma samples of healthy pregnant women (NC-exos), pregnant women with APS (APS-exos) and cell supernatant by 3000 × g centrifugation at 4°C for 5 min to remove cells and cellular debris, followed by 10000 × g centrifugation at 4°C for 60 min (Avanti J-26S XP). The supernatant was combined with phosphate buffered saline (PBS, Biological Industries, Israel) and then centrifuged at 100000 × g at 4°C for 120 min (OptimaXE-90 Ultracentrifuge). The exosome pellets were resuspended in PBS and stored at −80°C.

### Transmission Electron Microscopy (TEM)

To ascertain the purity of isolated exosomes, the samples (approximately 10 µg/ml) were fixed in 1% (final concentration) glutaraldehyde in 0.1 M phosphate buffer, pH 7.4. After attachment to TEM grids, the exosomes were negatively stained with 1% uranyl acetate. Images were taken from different regions of the grid.

### Nanoparticle Tracking Analyzer (NTA)

The sample cells were rinsed with deionized water, and the analyzer was calibrated with polystyrene microspheres (110 nm). After the sample cells were washed with PBS, samples were diluted in PBS and analyzed in triplicate.

### Lentiviral Transduction

Lentivirus was provided by the Hanyin Biotechnology Limited Company (Shanghai). The carrier component was CMV-MCS-EF1ɑ-ZsGreen1-PGK-puro. To demonstrate the effects of APOH protein, we transduced human embryonic kidney cells (293T) cells with lentivirus overexpressing APOH (APOH-293T) or empty lentivirus (N-293T) as a control. After transduction, the APOH-exos and control (N)-exos were isolated from cell culture medium.

### Immunofluorescence Microscopy

Human umbilical vein endothelial cells (HUVECs) were inoculated on glass-bottom dishes and cultured to 50% confluence. 1,1’-dioctadecyl-3,3,3’,3’-tetramethylindocarbocyanine perchlorate (Dil, Thermo Fisher) was added to the dishes to stain exosomes overnight. The cells were then fixed in 4% paraformaldehyde for 10 min and permeabilized in 0.1% Triton X-100 in PBS for 10 min at room temperature. After being washed and resuspended in PBS, the cells were incubated with phalloidin-FITC (Sigma, Darmstadt, Germany) for 30 min, washed in PBS, stained with 4′,6-diamidine-2-phenylindole (DAPI, Sigma) for 10 min, and washed again in PBS. Finally, the cells were placed on slides and covered with Fluoromount-GTM water-soluble compound. The slides were observed by confocal microscopy on an inverted microscope (TCS SP8; Leica, Weizlar), and fluorescence images were captured.

### Isolating, Grouping and Processing of HUVECs

Residual blood was washed out with PBS, and the umbilical vein cavity was digested in type I collagenase (Sangon Biotech) diluted to 0.8% in Hanks balanced saline solution at 37°C for 15–20 min. Subsequently, the contents were quickly rinsed in a 50 ml centrifuge tube, centrifuged at 1500 × g for 3 min, and transferred to a 3 cm culture dish. After 24 h of cultivation, the medium was replaced. The third to sixth generations of HUVECs were used for all experiments.

### Cell Culture

Using the conventional culture method (i.e., 37°C, 5% CO_2_ and 100% relative humidity in an incubator), the HUVECs were incubated in endothelial culture medium containing 5% fetal bovine serum and 1% endothelial cell growth factor (ScienCell), and the 293Ts were incubated in high-glucose Dulbecco’s modified eagle’s medium (DMEM, HyClone) and placed in saturated humidity culture conditions. The 293Ts were incubated in high-glucose DMEM containing 5% fetal bovine serum (HyClone) until they reached 80%–90% confluence. Then, the 293Ts were cultured for 48 h in high-glucose DMEM containing 2% BSA, and the supernatant was collected.

### Cell Proliferation, Migration and Tube Formation Assays

Proliferation, migration, and tube formation abilities of HUVECs were routinely evaluated. In brief, 3 × 10^3^ cells per well were plated in 96-well plates, then different doses of exosomes were added. Cell numbers were assessed using cell counting kit-8 at 450 nm (Thermo Fisher Scientific). For migration, 8 × 10^4^ cells were seeded in the upper chamber of a transwell chamber, and exosomes were added to the lower chamber. Following a 16 h incubation, hematoxylin staining was performed for each chamber. Migrated cells were counted in a transwell chamber (Corning BioCoat, USA). To quantitate *in vitro* tube formation, plates were coated with Matrigel (Corning BioCoat, 35420) substrate. HUVECs in serum-free endothelial cell medium were added with different doses of exosomes and photographed using an inverted microscope within 6 h. Images were taken at 40/100× magnification by optical microscopy. The above process was repeated three times, and the results were counted with ImageJ software.

### Protein Extraction and Quantification

Exosomes collected form each group were added to a 1.5 ml centrifuge tube containing 100 μl of Trizol and placed on ice for 30 min, ultrasonicated on ice with a high-intensity sonicator (Sonicator 4000), and centrifuged at 10000 × g (Centrifuge 5424R) for 15 min at 4°C. Finally, the supernatant was collected and analyzed with a BCA assay kit according to the manufacturer’s instructions.

### Western Blot Identification

Exosomes suspended in PBS were prepared using RIPA lysis buffer (Beyotime Biotechnology, Haimen, China) supplemented with a protease inhibitor cocktail and phosphatase inhibitor cocktail (Roche, Branford, CT, USA). Proteins were separated by SDS-polyacrylamide gel electrophoresis and transferred to a polyvinylidene fluoride membrane (Immobilon-Psq, Millipore). The membrane was removed and placed in an incubation cassette containing 5% BSA blocking solution for 2 h at room temperature. The primary antibody was diluted according to the manufacturer’s instructions and incubated with the membrane overnight at 4°C on a shaker. Protein band gray value analysis was performed with ImageJ software. The following primary antibodies were used for western blotting: APOH (Abcam), Erk (Cell Signaling Technology), P-Erk (Cell Signaling Technology), p38 (Cell Signaling Technology), P-p38 (Cell Signaling Technology), GAPDH (Cell Signaling Technology), CD63 (System Biosciences), CD81 (System Biosciences), CD9 (System Biosciences), HSP70 (System Biosciences), Calnexin (Cell Signaling Technology) and Grp94 (Cell Signaling Technology).

### Quantitative Proteomics Analysis With Total-Protein Tandem Mass Tag (TMT) Labeling

Exosome samples were processed for TMT-labeled quantitative proteomics analysis by Jingjie PTM BioLab (Hangzhou, China). First, the protein sample concentration was determined with a Pierce BCA protein assay kit (Thermo Fisher Scientific). After trypsin digestion, the peptides were desalted with Strata X C18 (Phenomenex) and vacuum freeze-dried. The tryptic peptides were fractionated by high-pH reverse-phase HPLC using an Agilent 300 Extend-C18 column (5 µm particles, 4.6 mm ID, 250 mm length). The peptides were dissolved in 0.1% (v/v) formic acid then loaded onto an EASY-nLC 1000 UPLC system. The peptides were treated with a nanospray ionization source followed by tandem mass spectrometry (MS/MS) in a Q ExactiveTM Plus (Thermo) coupled online to the UPLC. The resulting MS/MS data were processed by the Maxquant search engine (v.1.5.2.8).

### Animal Model Establishment and Processing

Female BALB/c nude mice (aged 5 weeks, experimental animal license number m20190601) were randomly divided into four groups and each group was injected with APS-exos (100 μg/ml/each), NC-exos (100 μg/ml/each), N-exos (50 μg/ml/each) or APOH-exos (50 μg/ml/each) *via* the caudal vein once a week for three continuous weeks. Seven days after the final treatment, the mice were mated overnight with male mice. Mating was determined by the appearance of a vaginal plug the following morning. On days 12–14 after mating, all female mice were sacrificed by cervical dislocation. The uteri were removed, and the total numbers of implanted embryos and resorbed embryo-fetal sites were counted. The placental tissue was collected for paraffin sectioning and hematoxylin and eosin (H&E) staining.

### Evaluation of Pregnancy Outcomes

The embryo loss rate (ELR) was calculated as the ratio of resorbed embryos/fetuses to the number of all fetuses. The mean number of embryos implanted (MNEI) was calculated as the ratio of total fetuses to the total number of mice.

### Hematoxylin and Eosin Staining

Tissues were fixed with 4% paraformaldehyde for 48 h and processed by conventional procedures. For paraffin-embedded tissue, sections of 3 to 5 μm thickness were cut from paraffin-embedded tissues, mounted on poly-L-lysine-coated slides, deparaffinized in xylene, dehydrated in alcohol, and stained with H&E. Pathological changes in the placenta were observed by H&E staining using electron microscopy (PHILIP CM-120). The vascular condition was evaluated independently by a pathologist (Dr. He).

### Statistical Analysis

All data were analyzed with GraphPad Prism 6.0 from GraphPad Software (San Diego, USA). The t-test was used to compare data between two groups, the nonparametric test was applied to non-normally distributed data, Chi-square test and Fisher’s exact test were applied for proportions. P-values <0.05 were considered to be statistically significant. *P < 0.05; **P < 0.01; ***P < 0.001; ns = not significant.

## Results

### Plasma Exosome Isolation and Characterization

We adopted the 2006 International Consensus Statement on the Update of the Classification Standards for APS (17). The clinical features of healthy early pregnancy patients and early pregnancy patients with APS are shown in **Table 1**. There were no significant differences in maternal age (ranged from 25 to 35 years old) or gestational age (gestational age 6–8 weeks) between the two groups. The early pregnancy patients with APS were those with a history of abortion and positive laboratory test results for aPLs. The healthy early pregnancy patients were those with no history of abortion and negative laboratory test results for aPLs.

**Table 1:** The clinical features of healthy early pregnancy patients and early pregnancy patients with antiphospholipid syndrome (APS).

	NC (Mean ± SEM)	APS (Mean ± SEM)
Number	6	6
Maternal age (years)	28.33 ± 1.764	27.83 ± 0.703
Gestation age (days)	42.33 ± 2.951	41.00 ± 2.309
Abortion times	0	2.167 ± 0.167
Antiphospholipid antibody		
Anti-APOH IgM antibody	Negative	52.92 ± 4.565
Anti-APOH IgG antibody	Negative	Negative
Anti- cardiolipin	Negative	Negative
Lupus anticoagulant	Negative	Negative

**Figure 1:**
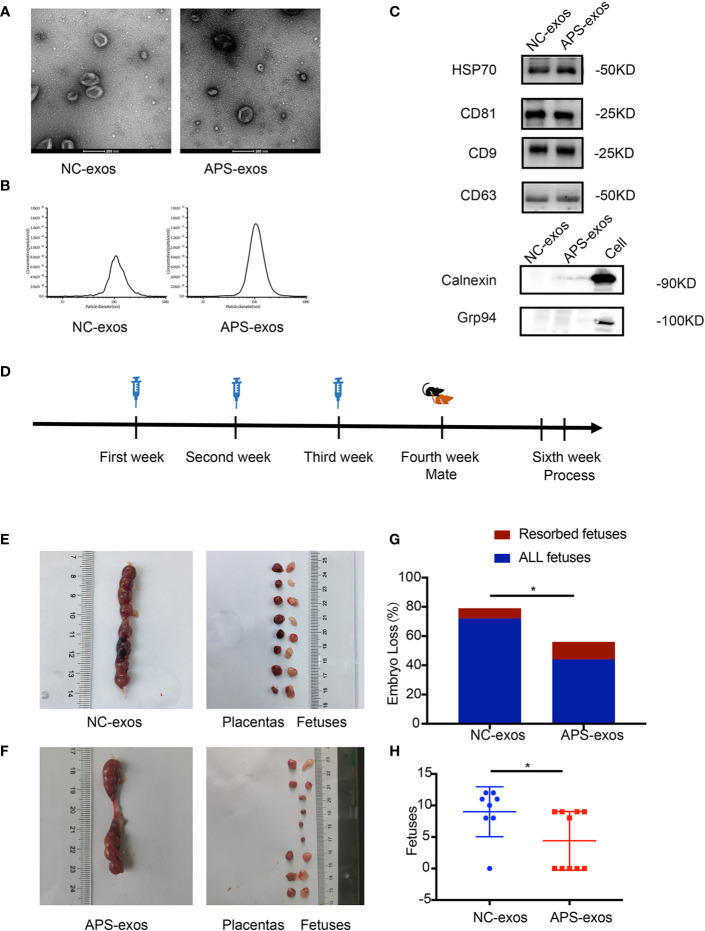
**(A)** Transmission electron microscopy (TEM) images of collected exosomes. Scale bars in the images represent 200 nm. **(B)** Diagram of the particle size distribution obtained by nanoparticle tracking analysis. **(C)** Confirmation of marker proteins in the exosome (exos) preparations. Western blotting of samples for HSP70, CD81, CD9, CD63, Calnexin and Grp94. **(D)** The mice were injected with antiphospholipid syndrome (APS)-exos (100 μg/ml/each) or normal control (NC)-exos (100 μg/ml/each) *via* the caudal vein once a week for 3 continuous weeks. Seven days after the final immunization, the treated female BALB/c mice were mated overnight with male mice. Mating was detected by the appearance of a vaginal plug the following morning. On days 12–14 after mating, all female mice were sacrificed. **(E, F)** Establishment of the abortion model. Typical uterus and embryos from a mouse treated with APS-exos. **(G)** The bar shows the percentage of embryo-fetal loss in the different groups. APS-exos increased the abortion rate of mice (NC-exos, n = 7 and APS-exos, n = 5, *P = 0.038 by chi-square test). **(H)** The scatter plot shows the pregnancy rates in different groups. APS-exos reduced the pregnancy rate. (NC-exos, n=8 and APS-exos, n =10, *P = 0.029 by Mann–Whitney test).

Purified exosomes were characterized (**Figures 1A–C**). By transmission electron microscopy, circular vesicular particles with a diameter of approximately 100 nm and lipid bilayer membranes were observed (**Figure 1A**). The NTA technique determined particle size distribution and particle concentration of the exosomes. As shown in **Figure 1B**, the particle diameter plot showed a single-peak healthy distribution with peaks at approximately 106.8 nm and 106.7 nm, primarily ranging from 30–150 nm. The particles in the sample were uniformly dispersed. Western blotting identified marker proteins CD9, CD63, CD81 and HSP70 on the serum exosome surface (**Figure 1C**), whereas intracellular proteins Calnexin and Grp94 were not detected. There was no difference in exosomal morphology between NC-exos and APS-exos.

### APS-Exos Cause an Antiphospholipid Syndrome-Like Phenotype in Pregnant Mice

**Figure 2:**
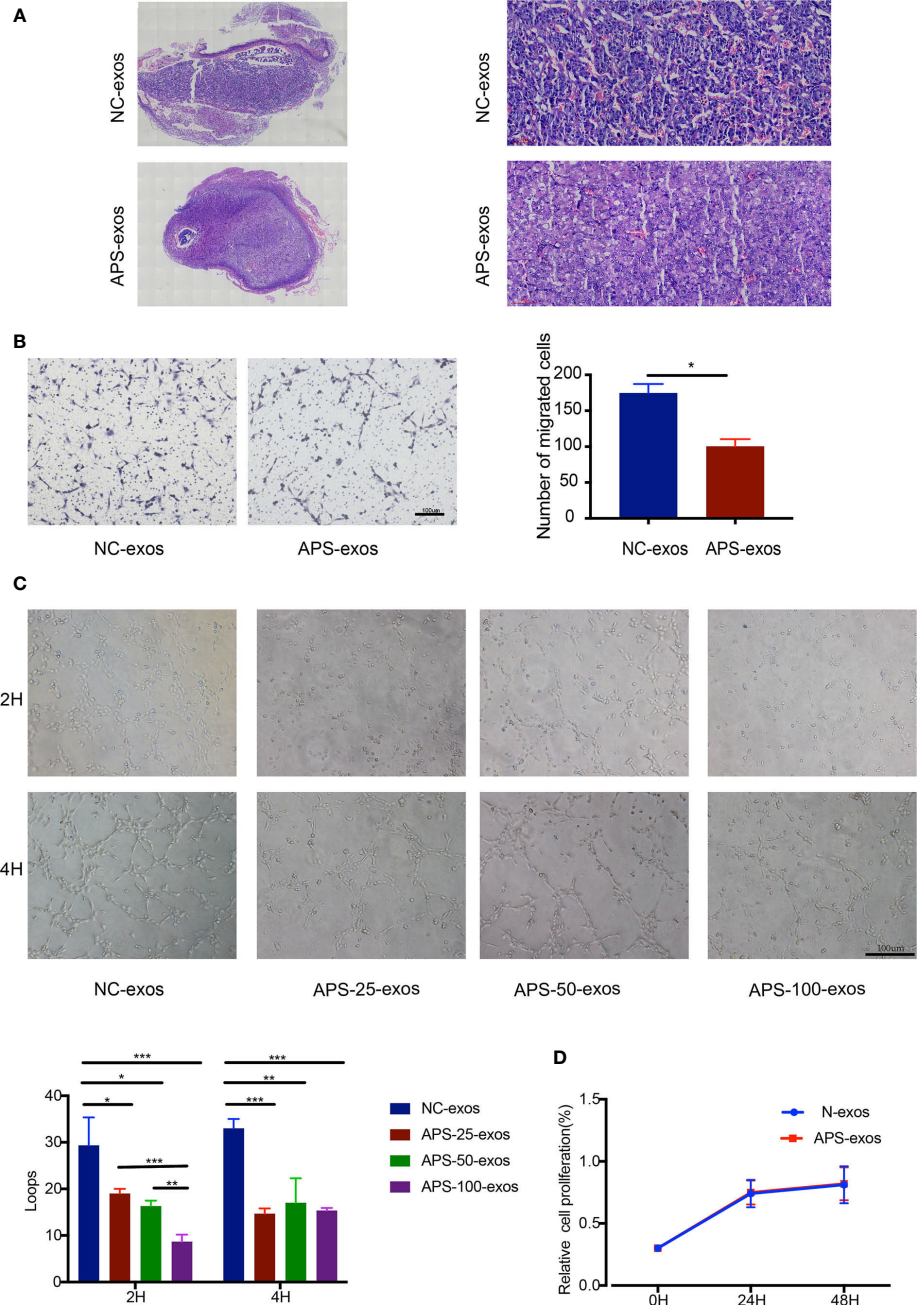
**(A)** Pathological analysis of hematoxylin and eosin (H&E)-stained placental tissue. H&E staining of placental tissue from the antiphospholipid syndrome-exosomes (APS-exos) and normal control (NC)-exos mouse model analyzed by light microscopy (2× and 20×). Placental blood vessels in mice treated with APS-exos compared with those treated with NC-exos. **(B)** After incubation with APS-exos or N-exos (50 μg/ml) for 16 h, human umbilical vein endothelial cell (HUVEC) migration was analyzed. *P = 0.029 by Paired t test. **(C)** After incubation with NC-exos (50 μg/ml) or APS-exos (25 μg/ml, 50 μg/ml, 100 μg/ml) for 2 h and 4 h, angiogenesis of HUVECs was analyzed. *P < 0.05; **P < 0.01; ***P < 0.001 by t test. **(D)** After incubation with APS-exos/NC-exos (50 μg/ml) for 24h and 48h, HUVEC proliferation was analyzed by the cell counting kit-8 assay. *P = 0.183 by Paired t test. All data shown as mean ± S.D.

To investigate the effect of APS-exos on embryo resorption, all pregnant mice were sacrificed 12–14 days after plug formation, and embryos and placentas were collected for analysis (**Figure 1D**). Typical embryos and placenta from a mouse treated with NC-exos and APS-exos are shown in **Figures 1E, F**. We found that the resorption rate of the APS-exos group (27.27%) was significantly higher than that of the control group (9.72%), with representative fetal reabsorption observed by the chi-square test (**Figure 1G**, **Table 2**). The MNEI of the APS-exos group was significantly lower than that of the control group (4.4 versus 9.0, respectively, **Figure 1H**). By electron microscopy, the placental angiogenesis of mice treated with APS-exos was decreased, but the difference was not significant (**Figure 2A**). These results indicate that exposure to APS-exos may result in APS-like adverse reproductive outcomes.

**Table 2:** Embryo loss rate of antiphospholipid syndrome-exosomes (APS-exos) and control (NC)-exos immunized mice (P = 0.038).

Data analyzed	All fetuses	Normal fetuses	Resorbed fetuses	Embryo Loss Rate	chi-square test	
NS-exos	72	65	7	9.72%		
APS-exos	44	32	12	27.27%	χ^2^ = 4.28	P = 0.038
Total	116	97	19			

### APS-Exos Reduced Endothelial Function

To investigate the effect of plasma exosomes on endothelial cells in early pregnancy patients with APS, we performed proliferation, migration, and tube formation assays. APS-exos had no effect on HUVEC proliferation, but significantly inhibited HUVEC migration and impaired tube formation (**Figures 2B–D**).

### Quantitative Proteomics Analysis Identified APOH as a Key Target Protein

**Figure 3:**
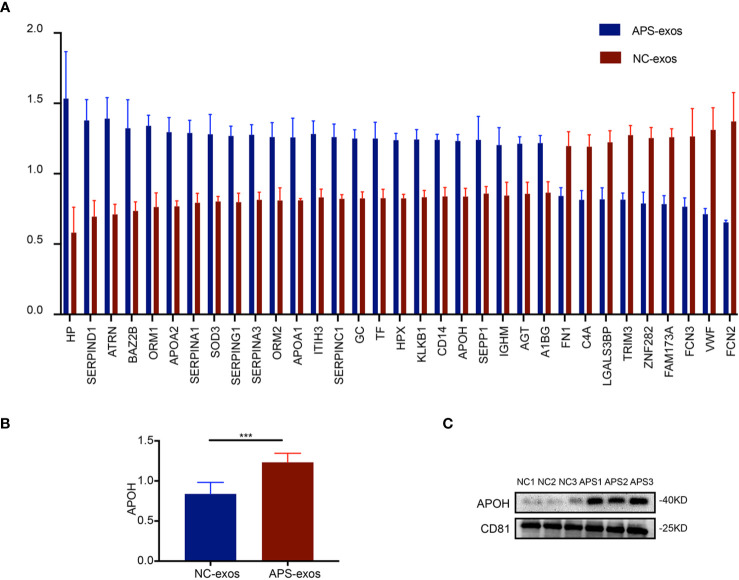
Proteomic analysis results. **(A)** A total of 293 proteins were identified from the two groups. Twenty-seven upregulated proteins and nine downregulated proteins were identified by quantitative proteomics tandem mass tag analysis of antiphospholipid syndrome-exosomes (APS-exos) and normal control (NC)-exos. Mean ± SEM (36 proteins were significantly different, P < 0.05 by t test). **(B)** The difference in APOH expression between the two groups shown by proteomic analysis (P = 0.000 by t test). **(C)** Clinical validation showed that the level of APOH was significantly higher in APS-exos than that in NC-exos. “***” means P = 0.000 by t test.

To determine the difference between APS-exos and NC-exos, we carried out a quantitative proteomics analysis of whole protein TMT markers. A total of 293 proteins were identified from the two groups, including 261 quantified proteins. Values >1.4-fold of the baseline value were chosen as significant upregulation, and <1/1.4-fold was chosen as significant downregulation. Twenty-seven upregulated proteins and nine downregulated proteins were identified. A summary of all the differentially expressed proteins identified in this study are shown in **Figure 3A**. We examined the APOH protein, as it is a major antigen of APS (1, 5). Difference in APOH levels between the two groups were shown by proteomic analysis (**Figure 3B**). Furthermore, the differential protein expression found by TMT analysis was confirmed *via* western blotting, which indicated that APOH was significantly higher in APS-exos than that in NC-exos (**Figure 3C**).

### Gene Ontology (GO) and KEGG Pathway Analysis

**Figure 4:**
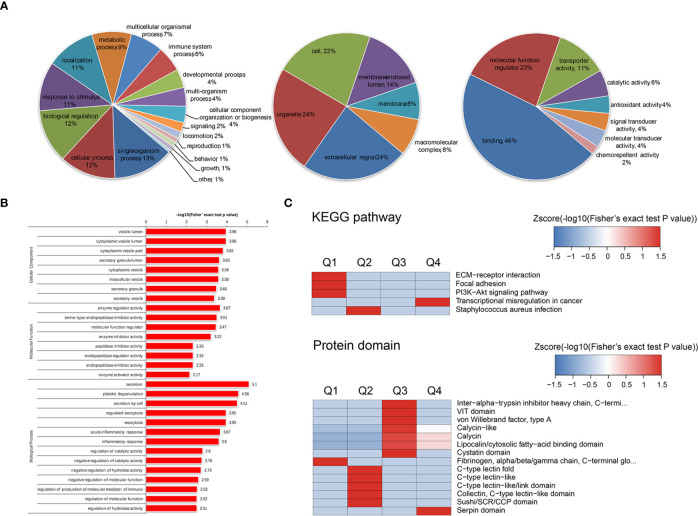
**(A)** Gene Ontology (GO) annotation was derived from the UniProt-GOA database. GO enrichment analysis of upregulated proteins in the biological process (left), cellular component (mid), and molecular function (right) categories. **(B)** We enriched and analyzed the entries in GO annotations except for the first three categories (biological process, cellular component and molecular function). **(C)** The clustering method is based on the p value of Fisher’s exact test obtained from enrichment analysis. The hierarchical clustering method was used to gather relevant functions in different groups and draw them as a heat map. The horizontal of the thermogram represents the enrichment test results of different groups, and the vertical represents the description of differential enrichment related functions (KEGG pathway, protein domain). The different proteins and the color blocks corresponding to the functional description in different groups indicate the degree of enrichment. Red indicates a strong degree of enrichment, while blue indicates a weak enrichment. KEGG pathways with significant enrichment of differentially expressed proteins [antiphospholipid syndrome-exosomes (APS-exos) versus normal control-exos) (above). Cluster analysis heat map based on protein domain enrichment (below).

The APS-exos- and NC-exos-induced differentially expressed proteins were categorized into GO annotation classes. Based on the above data, we carried out a systematic bioinformatics analysis of proteins containing quantitative information, including protein annotation, functional classification, functional enrichment and clustering analysis based on functional enrichment (**Figures 4A–C**). The upregulated and downregulated proteins are mainly involved in binding, molecular function regulation, catalytic activity, transporter activity and other functions. Differentially expressed proteins were divided into four groups (Q1 to Q4) according to their differential multiples: Q1 (0 < ratio ≤ 1/1.4), Q2 (1/1. < ratio ≤ 1/1.2), Q3 (1.2 < ratio ≤ 1.4) and Q4 (ratio > 1.4). We then enriched the KEGG and protein domains in each Q-group, and a cluster analysis was performed to find the correlation of protein functions with different expression multiples.

### APOH-Exos Caused an Antiphospholipid Syndrome-Like Phenotype in Pregnant Mice

**Figure 5:**
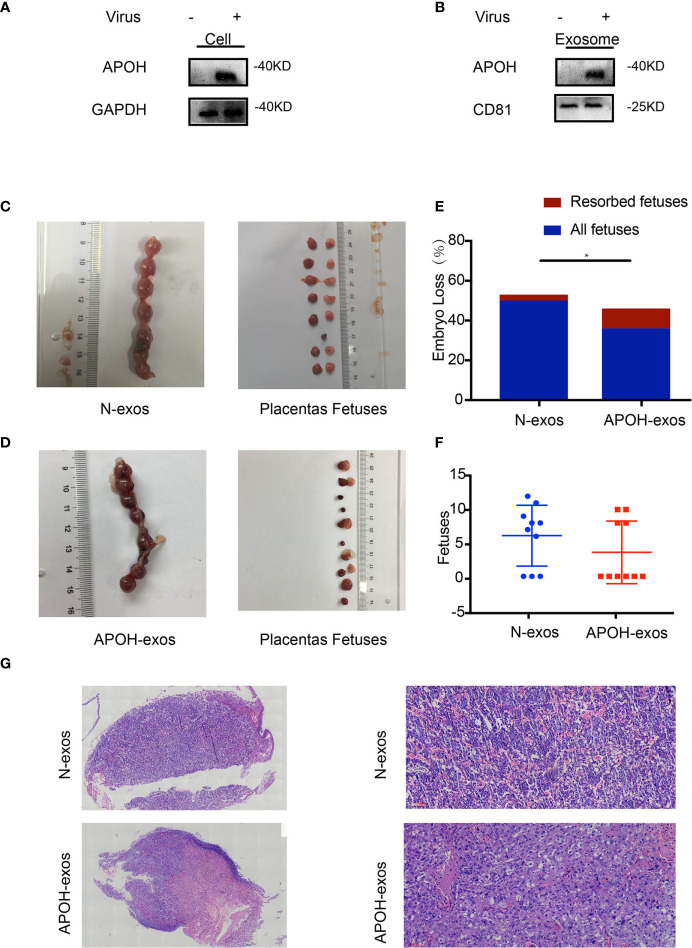
**(A, B)** Apolipoprotein H (APOH) lentivirus was transduced into 293T cells to enhance APOH expression. APOH overexpression and control (NC) lentiviral vectors were constructed. The cells and exosomes [N-exosomes (exos) and APOH-exos] were examined by western blotting. **(C, D)** Establishment of the abortion model is shown in **Figure 1D**. Typical embryos and uterus from a mouse treated with APOH-exos and NC-exos (50ug/ml/each). **(E)** APOH-exos increased the abortion rates of mice (NC-exos, n = 7 and APOH-exos, n = 4; *P = 0.012 by Fisher’s exact test). **(F)** No significant difference in pregnancy rate between the two groups. (NC-exos, n =10 and APS-exos, n =10; *P = 0.319 by Mann–Whitney test). **(G)** Pathological analysis of hematoxylin and eosin-stained placental tissue from the APOH-exos mouse model by light microscopy (2× and 20×). The placental blood vessels and the vascular thrombus in mice treated with APOH-exos compared with those in mice treated with N-exos.

To investigate the effect of APOH on endothelial cells, we constructed APOH-overexpressing lentiviral vectors and transduced the APOH lentivirus into 293T cells to enhance APOH expression (APOH-293T). Compared with the NC vector (N-293T), the APOH vector increased the expression of APOH protein in 293T cells and the secreted exosomes (**Figures 5A, B**).

To identify the effects of APOH-exos on embryo resorption, all pregnant mice were sacrificed 12–14 days after plug formation, and the embryos and placenta were sampled for further analysis (**Figures 5C, D**). We found that the embryo-fetal resorption rate for the APOH-exos group (27.78%) was significantly higher than that of the control group (4.91%); representative reabsorption is shown in **Figure 5E** and **Table 3**. There was no significant difference in MNEI levels between the APOH-exos and control groups (**Figure 5F**). By electron microscopy, placental angiogenesis of mice treated with APOH-exos was not significantly different compared with control mice (**Figure 5G**).

**Table 3:** Embryo loss rate of Apolipoprotein H-exosome (APOH-exos) and control (N)-exos immunized mice (P = 0.012).

Data analyzed	All fetuses	Normal fetuses	Resorbed fetuses	Embryo Loss Rate	Fisher's exact test
N-exos	50	47	3	4.91%	P = 0.012
APOH-exos	36	26	10	27.78%	
Total	86	73	13		

### Inhibition of HUVEC Migration and Tube Formation by APOH-Exos

**Figure 6:**
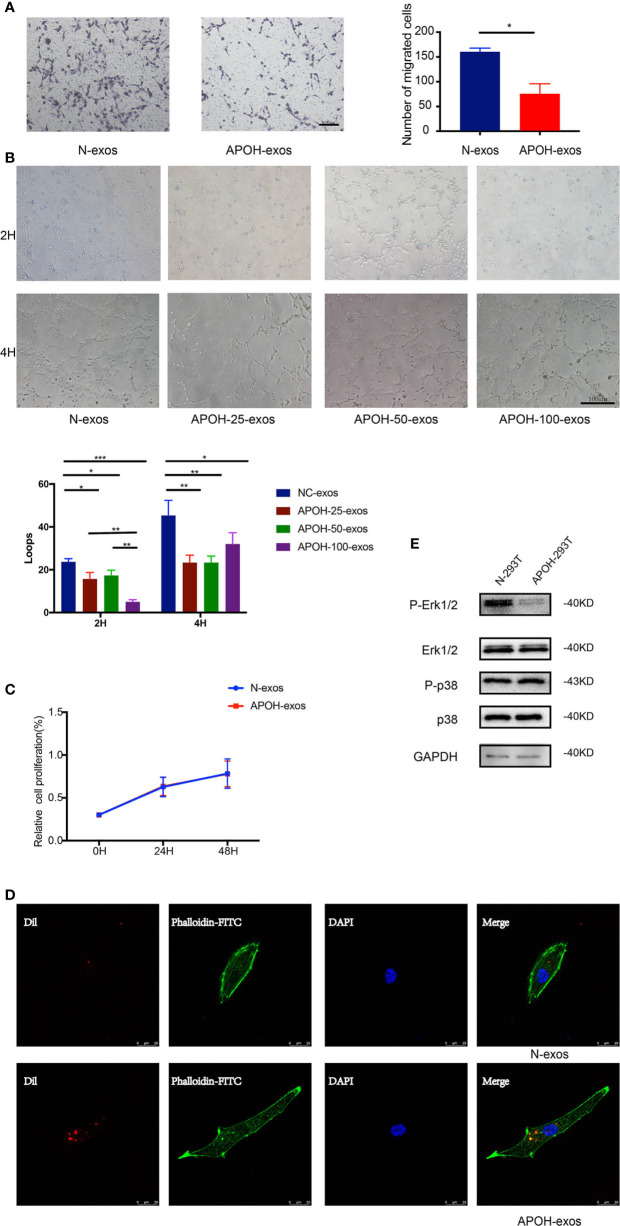
**(A)** After incubation with 25 μg/ml APOH-exosome (exos) or 25 μg/ml control (N)-exos for 16 h, human umbilical vein endothelial cell (HUVEC) migration was analyzed. Mean ± SD (*P = 0.014 by Paired t test). **(B)** After incubation with N-exos (50 μg/ml) or APOH-exos (25 μg/ml, 50 μg/ml, 100 μg/ml) for 2 h and 4 h, HUVEC angiogenesis was analyzed. Mean ± SD (*P < 0.05; **P < 0.01; ***P < 0.001 by t test). **(C)** After incubation with APOH-exos/N-exos (25 μg/ml) for 24h and 48h, HUVEC proliferation was analyzed by the cell counting kit-8 assay. Mean ± SD (*P = 0.183 by Paired t test). **(D)** The binding and endocytosis of APOH-exos/N-exos in HUVECs was observed by laser scanning confocal microscopy. **(E)** After 16 h of incubation of HUVECs with APOH-exos, the levels of Erk 1/2, P-Erk 1/2, p38 and P-p38 were determined by western blotting.

To determine the relationship between APOH-exos and HUVEC function, we performed proliferation, migration, and tube formation assays. APOH-exos had no effect on proliferation but significantly inhibited HUVEC migration and reduced tube formation (**Figures 6A–C**).

### APOH-Exos Inhibited the Phosphorylation of Erk1/2

To further investigate the mechanism of APOH-exos actions, APOH-exos were observed by laser scanning confocal microscopy (**Figure 6D**). The binding and endocytosis of APOH-exos were observed in HUVECs. After 16 h of incubation of HUVECs with APOH-exos, the level of phospho-extracellular signal-regulated kinase 1/2 (P-Erk1/2, **Figure 6E**) was decreased, whereas the levels of Erk1/2, p38 and phospho-p38 (P-p38) were the same. APOH may inhibit HUVEC angiogenesis through the P-Erk signaling pathway.

## Discussion

Exosomes carrying proteins and other agents play a role in intercellular communication. There is increasing evidence showing exosomes play a critical role in pregnancy (18, 19). APS is a unique multiple-system disorder that leads to thrombosis and/or pregnancy complications. Here, we provide evidence that exosomes containing APOH protein from early pregnancy patients with APS can induce vascular endothelial dysfunction and facilitate adverse reproductive events.

Angiogenesis is a pivotal process in embryo development and tissue growth, but also acts as a key player in many pathological conditions. Angiogenesis includes several steps characterized by endothelial cell function, such as migration and lumen formation (20). APLs in APS can activate endothelial cells and exosome release. Studies have shown a significant increase in the level of exosomes in patients with APS, but the relationship between exosomes and thrombosis and obstetric events has not been confirmed (16). In this study, we revealed that the exosomes of patients with APS can cause adverse reproductive events in mice. We found that APS-exos increased the abortion rate and reduced the successful pregnancy rate of mice. At present, the reason for the APS-exos-induced reduction of successful pregnancy in mice remains undetermined. We found that the abortion rate was increased in the APS-exos mouse model, and we also confirmed that exosomes from patients with APS induced HUVECs dysfunction. Therefore, we hypothesized that some proteins in exosomes of APS patients lead to an increased abortion rate by inhibiting angiogenesis. To explore the exosome proteins involved in APS-like actions, we compared proteomic profiles using high-resolution TMT technology (21).

The APOH protein levels found by protein mass spectrometry attracted our attention. APOH is an important antigen of APS, and the antigen-antibody complex of antiphospholipid antibodies and APOH increases the affinity for negatively charged phospholipids on the cell surface, thereby activating endothelial cells, mononuclear cells and platelets (1, 5, 22, 23). Past work showed angiogenesis was significantly higher in APOH gene-deficient mice compared with healthy mice (24). Treatment with APOH has been shown to induce experimental antiphospholipid syndrome in naive mice and can induce APS-like characteristics in mice (23).

In the current study, 293T cells overexpressing APOH by lentivirus transduction produced exosomes that also contained high levels of APOH. We found that the abortion rate increased in APOH-exos-treated mice. In vitro experiments showed that APOH-exos can also induce endothelial dysfunction and affect angiogenesis. Previous studies have shown that aPLs from patients with APS activated cells *via* the p38 and Erk pathways (25–27). We further explored the underlying mechanism and found Erk1/2 phosphorylation was significantly inhibited in the APOH-exos group compared with the control group, whereas Erk1/2, p38 and P-p38 levels were equivalent in both groups, suggesting that the P-Erk signaling pathway was inhibited by APOH. P-Erk is a member of mitogen-activated protein kinase (MAPK) signaling pathway, and plays an important role in cell growth, migration and angiogenesis (25–33). APOH-exos may inhibit P-Erk1/2, then inhibit cell migration and tube formation, which means the inhibit of angiogenesis. Our data suggest that APOH protein in exosomes from APS patients might inhibit angiogenesis through the P-Erk pathway, resulting in an increased rate of abortion. Importantly, APOH-exos can be directly transferred into HUVECs and inhibit endothelial cell migration and tube formation through the suppression of P-Erk, rather than activating the pathway by binding the antiphospholipid antibody and antigen to the surface of the phospholipid (34).

In conclusion, we propose that APS-exos may be a key factor in the pathogenesis of APS and that APOH may be a key protein that impairs vascular biological function. Additional studies are required to determine the mechanisms by which exosomal APOH induces vascular dysfunction and to investigate the potential use of specific inhibitors to block relevant pathways to treat APS.

## Data Availability Statement

All the data and techniques within the article are available from the corresponding authors upon reasonable request.

## Ethics Statement

The studies involving human participants were reviewed and approved by Ethics Committee of the Shanghai First Maternal and Infant Hospital affiliated with Tongji University (KS18128). The patients/participants provided their written informed consent to participate in this study. The animal study was reviewed and approved by Ethics Committee of East China Normal University (m20190601). Written informed consent was obtained from the individual(s) for the publication of any potentially identifiable images or data included in this article.

## Author Contributions

All authors listed have made a substantial, direct, and intellectual contribution to the work, and approved it for publication.

## Funding

This work was supported by Shanghai Science and Technology Commission Innovation Plan (No. 17411951600), the National Natural Science Foundation of China (No. 81672574, No. 81804128, No. 81972438) and Shanghai Key Clinical Specialty Programme (No. 2017ZZ02015).

## Conflict of Interest

The authors declare that the research was conducted in the absence of any commercial or financial relationships that could be construed as a potential conflict of interest.
